# Unlocking Nanocarriers for the Programmed Release of Antimalarial Drugs

**DOI:** 10.1002/gch2.201600011

**Published:** 2017-01-30

**Authors:** Amir Reza Bagheri, Seema Agarwal, Jacob Golenser, Andreas Greiner

**Affiliations:** ^1^ Macromolecular Chemistry Bavarian Polymer Institute University of Bayreuth Universitätsstraße 30 95440 Bayreuth Germany; ^2^ Department of Microbiology and Molecular Genetics The Kuvin Centre for the Study of Infectious and Tropical Diseases The Hebrew University of Jerusalem 91120 Jerusalem Israel

**Keywords:** drug carrier, electrospinning, malaria, programmed drug release

## Abstract

**A programmable release system** with wide range of release profiles of the antimalarial artemisone (ART) from fibrous nanocarriers (NFN) is presented. This is achieved following a new paradigm of using ART‐loaded NFN in infusion system of hydrophobic drug eluting nanocarriers, adapted to clinical applications. Very importantly, under these conditions ART did not degrade as it was observed in solution.

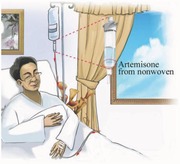

Malaria is one of the most threatening diseases which annually, causes the death of about 580 000 people (WHO, fact sheet 2014). Several drugs and drug delivery systems were tried in the past for prevention and treatment of malaria.^1^, ^2^, ^3^ In recent years, the therapy of this fatal disease was improved by artemisinin drugs.^4^, ^5^, ^6^ One promising artemisinin derivative is artemisone (ART), which shows profound therapeutic effects in mouse malaria models and against falciparum malaria.^7^, ^8^, ^9^ The relevance of the discovery of artemisinin and malaria research to humankind was emphasized by the 2015 Noble Prize to Youyou Tu. One of the challenges in the practical use of artemisinins as hydrophobic drugs, is the dosage that should be optimal for efficacy and lack of toxicity. A programmed/controlled drug release could be a solution for maintaining the optimal dosage of artemisinins for longer periods. The time‐dependent release of drug with or without external stimuli is referred as controlled release in general literature.^10^ In an ideal case, a delivery system should provide immediate an initial therapeutic drug concentration (burst dose) and then maintain this concentration for an extended period (sustained dose) depending upon the requirement. Further, the rate of sustained release of therapeutic concentration should match with the rate of the absorption by the body in order not to exceed the toxic level. This gets more complicated as physiological conditions, and therefore the rate of drug absorption varies from person to person. An ideal controlled release system for diseases like malaria should provide predictive release of one or more drugs, according to personal requirement. This should be independent of the environmental influence, in a reproducible manner, accessible and usable even under rural conditions, not requiring complicated technology, and also programmable according to the type and status of the infection. It is still an unresolved issue and impossible to achieve using presently known concepts of drug release such as inhalers, micro/nanoparticles, drug reservoir implants, and antibody–drug conjugates.^11^

The other main issues with artemisinins are low bioavailability, short half‐life, low solubility, and stability in aqueous medium.^12^, ^13^, ^14^, ^15^, ^16^ The bioavailability of artemisinin can be improved by using drug‐loaded liposomes as carriers^17^ but not in a predictive manner. The decomposition of artemisinins in the aqueous medium is critical as the physiological effect of the decomposition products is uncertain.^18^, ^19^ ART is rather expensive and cannot be stored in the form of liquid formulations for delivery purpose ruling out simple infusion formulations as one of the ways of controlled drug delivery. Although there are some efforts in the literature to provide controlled release of artemisinins in vitro from nanovesicles and solid lipid particles, but due to the complexity of the situation described above, the systems are far away from any practical utility.^20^ Moreover, these examples do not provide programmed release in which the amount can be controlled on demand. Due to the complexity of the problem and restrictions imposed by the nature of the drug, no simple solution is applicable. This is the general problem in the field of drug delivery where in vitro studies using various drug delivery carriers including nanofiber nonwoven (NFN) made by electrospinning^21^, ^22^, ^23^, ^24^ are indicative of applicability but not transferable to actual in vivo applications.

In an effort to solve this problem we decouple the programmed drug release from physiological environment. We suggest that the combination of the advantage of precise and reproducible programmed release from NFN in vitro with the drug delivery infusion system is very beneficial (**Figure** **1**). In our system, the control over drug release is achieved outside physiological environment by placing ART‐loaded NFN in the drip chamber of an infusion system which can release ART in adjustable manner. The ART‐loaded NFNs were additionally coated with the biocompatible polymer poly(*p*‐xylylene) (PPX) by chemical vapor deposition (CVD) to control the release profile and stability of the drug in infusion medium by confinement. Our drug‐delivery concept also takes care of the low solubility of ART in aqueous media, by using infusion medium with appropriate surfactant helping the release of the hydrophobic drug. The use of surfactants for increasing the solubility of hydrophobic drugs is well‐known besides many other concepts, such as particle‐size reduction (microsizing and nanosizing), salt formation, complexation with β‐cyclodextrins, and use of solid dispersions (freezing the crystalline drug in an amorphous state in a hydrophilic carrier matrix.^25^ The exact method depends on the type of drug used. Several of these methods are not applicable to ART because of its water and temperature instability.

**Figure 1:**
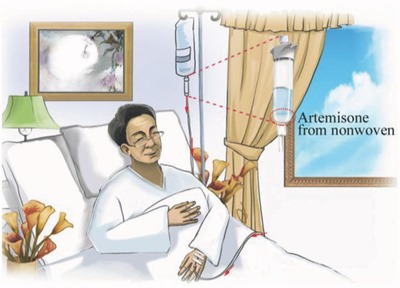
The depiction of the concept of programmed drug release from drug‐loaded polymeric nanofiber nonwoven carrier placed in the drip chamber of an infusion system.

The severe restrictions imposed on material choice in terms of biodegradability and biocompatibility for drug carriers for in vivo use is no more relevant in our case as the drug eluting membranes never come in contact with biological matter. Moreover, the release of ART could be easily programmed by the amount of drug‐loaded NFN, rate of infusion, and composition of the infusion medium. ART‐loaded NFN integrated into an infusion system provides a convenient tool, fulfilling the clinical demands toward a programmable and easy‐to‐use administration of ART with improved stability. The concept uses state‐of‐the‐art polymers, NFN formation technique, and infusion system making it available on a large scale. This system could be transferred to various other hydrophobic drugs and also can be used when drug combinations are needed, for example, artemisinin drug combinations (ACT).^26^, ^27^, ^28^, ^29^

First, we studied the loading of NFN with ART and its release properties without and with additional coatings in order to tailor the ART release profiles. The polymer used for the preparation of ART‐loaded NFN by electrospinning was the very well‐known block copolymer polycaprolactone (PCL)_16 500_‐*b*‐α‐hydroxy‐ω‐methoxy‐poly(ethylene glycol) (MPEG)_5000_ (PCL‐MPEG).^30^, ^31^, ^32^, ^33^ The polymer used for the additional coating was poly(xylylene) (PPX), which provides established biocompatible coatings.^34^, ^35^

PCL‐MPEG was prepared by ring‐opening polymerization as presented in details in the Supporting Information (Tables S1 and S2 and in Figures S1 and S2, Supporting Information, for molecular characterization). ART‐loaded NFNs were obtained by solution electrospinning of mixtures of PCL‐MPEG and ART in different compositions (0–20 wt% ART). Electrospinning is a state‐of‐the‐art method of making porous NFNs which has been utilized in the past for immobilization of several drugs by spinning a mixture of the template polymer and the corresponding medicines in the literature.^36^, ^37^

NFNs with smooth fiber surfaces and an average fiber diameter of 220 ± 65 nm (for samples with contents of 12.5 wt% ART) were obtained (**Figure** **2**a). The content of ART in NFN was quantified by ^1^H‐NMR and high‐pressure liquid chromatography (HPLC, see also Tables S3 and S4 and Figures S3–S7, Supporting Information). While NFN with 12.5 wt% of ART was homogenous (Figure 2a), NFN with a larger amount of ART (20 wt%) showed the formation of ART crystals (Figure 2b). The distribution of ART in NFN with 12.5 wt% ART was analyzed by energy dispersive X‐ray analysis (EDX) by tracing sulfur of ART. The EDX demonstrated the homogeneous incorporation of ART on the surface and cross section of NFN (Figure 2c,d). Consequently, the NFN samples loaded with 12.5 wt% ART were further studied in order to advance their practical application.

**Figure 2:**
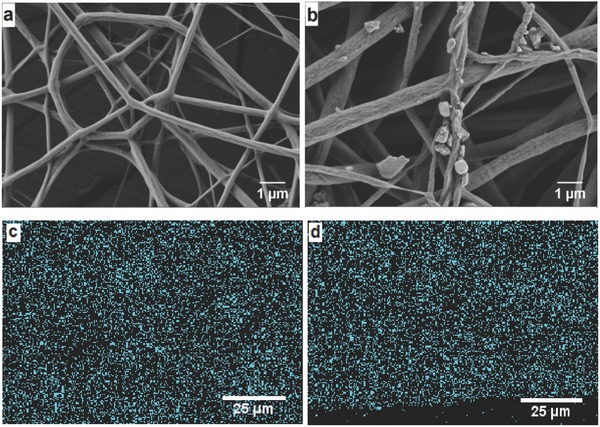
SEM and EDX images of ART loaded electrospun NFN. a) 12.5 and b) 20 wt% ART‐loaded NFN. c) The surface and d) cross section of ART‐NFN with the content of 12.5 wt% ART.

The in vitro ART release characteristics from NFN were studied by HPLC with PBS (phosphate buffered saline) of pH 7.4 as release test medium (**Figure** **3**a). The absolute release of ART within 5 h in PBS solution was only about 13.23 ± 0.036 wt%. The explanation for this relatively low and slow release of ART from NFN is its limited solubility in PBS: the maximum solubility of ART in PBS as determined by HPLC analysis was only 0.074 ± 0.002 mg mL^−1^. Even with an extended release time of several days (not shown here) a 100 wt% cumulative ART release was never observed. The solubility and release of ART were enhanced significantly to 1.79 ± 0.01 mg mL^−1^ with 1% w/v of sodium lauryl sulfate (SLS) in water as release medium. SLS acts obviously as a surfactant for ART as it is known to enhance solubility for other hydrophobic drugs.^38^ Consequently, fast and 100 wt% release of ART from NFN could be achieved by using the SLS medium (Figure 3a) in less than 1 h with more than 70% drug released in about 30 min.

**Figure 3:**
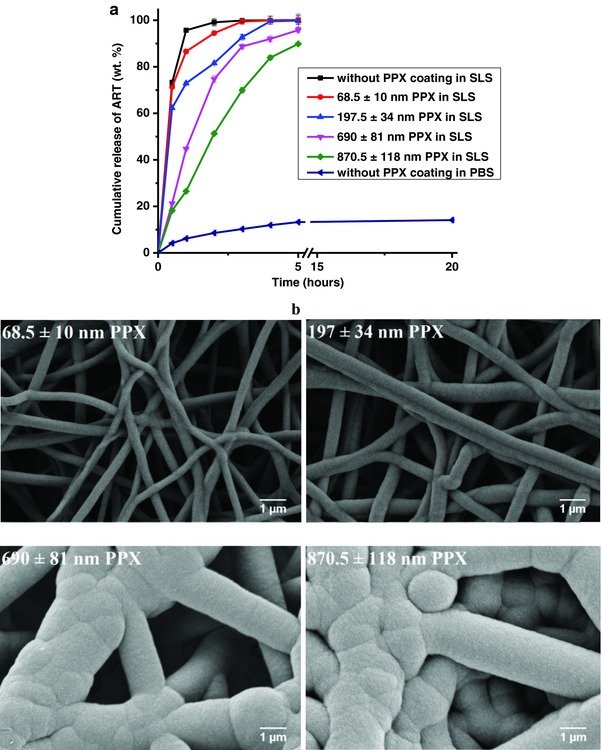
a) In vitro cumulative release of ART from uncoated and PPX coated NFN in an aqueous solution containing 1% SLS, ART content for all samples: 12.5 wt% and mean quantities of ART in all samples: 5.55 ± 0.34 mg. b) SEM images of PPX coated NFN with thicknesses from 68.5 to 870.5 nm used for in vitro release studies.

In order to overcome the burst release of ART and the problem of the instability of ART in contact with media, we applied the CVD coating of PPX of different thickness (68–870 nm) on the ART containing NFN (Figure 3b). An additional coating of drug carriers is a well‐established method for the retardation of drug release.^39^ PPX is a biocompatible polymer, which has been used successfully as a coating on films for the retardation of release of dexamethasone^40^ and on NFN for the retardation of enzyme release.^41^ The particular advantages of the CVD coating by PPX are very mild conditions (no solvent, no catalyst, room‐temperature processing) and the formation of conformal, pin‐hole free coatings. As expected, the PPX coating retarted the ART release and we observed a change from burst release (for uncoated fibers) to linear ART release profile depicting a delay that depends on the PPX coating thickness (Figure 3). The eluting drug was analyzed by HPLC for checking the stability. The instability of ART in aqueous media is one of the known problems giving degradation products of unknown toxicity and wastage of the valuable drug. This also limits its use in the form of liquid formulations for infusion, for example. We also carried out stability studies of ART powder to have the basis for comparison. In our studies, at 37 ± 0.5 °C almost 10 wt% of the ART powder was degraded in 1% w/v SLS at pH 7.4 ± 0.1 in ≈5 h. Whereas, ≈86 wt% of ART was decomposed in contact with SLS medium after 19 d (Figure S8, Supporting Information). The appearance of new signals in HPLC in combination with the decline of the signal for ART became evident by comparison of the HPLC traces of a fresh ART solution, with a solution after storage for 308 h (Figures S9 and S10, Supporting Information). Interestingly, the PPX‐coated samples showed 100 wt% release of ART without any degradation as proved by HPLC (not shown here) indicating the stability of ART. Apparently, ART is superior to other artemisinin derivatives in generation of clinically important metabolites (Haynes, personal communication).

The knowledge generated regarding in vitro drug release has very limited relevance for practical use because the scenario of drug release entirely changes in vivo. Some of the bottlenecks are: (1) the release of ART (same will be true for other hydrophobic drugs) would function only with media, which dissolve ART as good as SLS, which would not be possible in vivo; (2) effect of physiological media on the drug carrier will influence the release profile; and (3) programming the in vivo release of ART (i.e., controlling the amount of drug release according to the demand) from such drug carrier would not be possible and desires a different approach for enabling its application. Thus, we focused on a new and pervasive administration route for drug‐loaded nanocarriers based on the infusion therapy, which offers distinct advantages. The programming of drug release from ART‐loaded NFN is achieved in the infusion system. We want to emphasize here that infusion of ART and similar drugs by simply mixing in infusion medium is not possible due to their instability (please refer to arguments discussed in previous parts) in addition to the practical complexity of controlling the concentration according to demand.

Until now, implantation is almost the unique suggested administration route for the delivery of drug‐loaded NFN.^42^ Although implantation is seen as an efficient drug delivery method, practical in vivo application possesses different complications^43^, ^44^ that does not satisfy the need for simple and programmable drug administration. Our concept and setup is rather simple and could be manufactured under sterile conditions by placement of the ART‐loaded NFN in the drip chamber of the infusion set (Figure S11, Supporting Information). The release of different doses of ART could be programmed from burst release to retarded release and to different total release times (**Figure** **4**). Programming of ART release and dose was accomplished by the management of the flow rate, the formulation of the infusion medium, and the amount of ART in NFN placed in the drip chamber.

**Figure 4:**
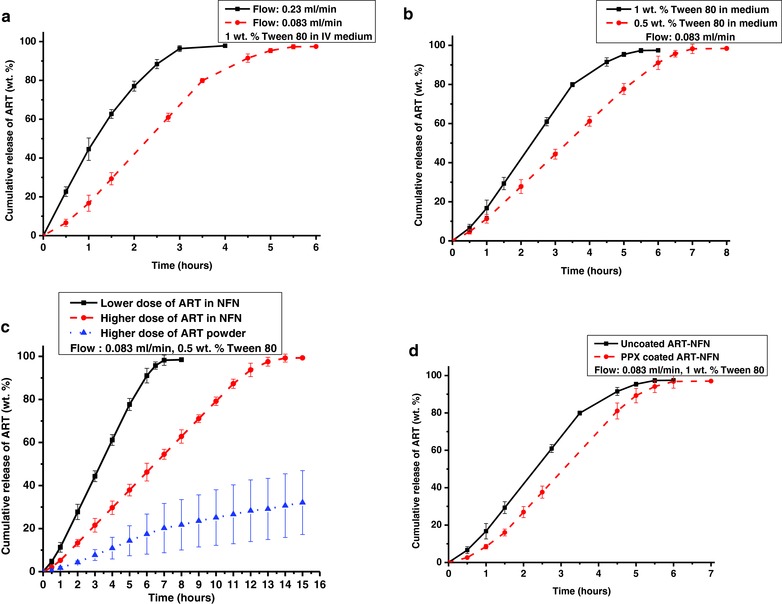
The programmed release of ART from NFN by infusion system, ART content for all formulation: 12.5 wt%. a) The effect of flow rates on ART release from NFN at two different flow rates of 0.083 and 0.23 mL min^−1^, the medium contains 1 wt% Tween 80. Mean amounts of ART in NFN: 5.49 ± 0.66 mg. b) The effect of the amount of Tween 80 on ART release from NFN at the adjusted flow rate of 0.083 mL min^−1^, but intravenous (IV) media include 0.5% and 1% Tween 80. Mean amounts of ART in NFN: 5.68 ± 0.39 mg. c) The effect of doses of drug on release behavior of ART from NFN, lower doses: 5.40 ± 0.10 mg drug and higher doses: 10.86 ± 0.10 mg, comaprison was also done by putting 10.86 mg of ART powder in the drip chamber (blue curve) and d) the effect of PPX coated layer with thickness of 68.5 ± 10 nm on ART release from NFN, mean amounts of ART in NFN: 6.42 ± 0.64 mg.

First of all, the amount of drug released and release profile, could be controlled by changing the flow rate of the infusion medium (0.9% w/v saline solution with 1 wt% biocompatible surfactant (Tween 80)^25^, ^45^) from the ART‐loaded NFN. The lower flow rate (0.083 mL min^−1^) avoided burst release, and the release of drug was completed in ≈5.5 h. Whereas, the higher flow rate (0.23 mL min^−1^) led to release of more than 20 wt% a drug already in the first 30 min and the drug release was completed in ≈3 h (Figure 4a).

Further, at a constant flow rate of the infusion medium, the drug release profile could be changed by using different concentrations of surfactant in the medium (Figure 4b). The amount of Tween 80 affects the solubility of the drug and hence the release profile. The maximum solubility of ART could be increased linearly by increasing the quantity of the surfactant (Tween 80) as shown in additional experiments (Figure S12, Supporting Information). Therefore, the maximum dose of ART eluting out could be well controlled by the amount of Tween 80 in the infusion medium, which is critical as overdoses of ART would be harmful.^16^, ^46^ The released drug could be sustained at a constant amount for more than 5 h on using the low concentrations of Tween 80 (0.5 wt%) at a flow rate of 0.083 mL min^−1^ besides avoiding the burst release. In this case, a relatively linear increase in cumulative release curve from 1 to 6 h could be obviously observed (Figure 4b). The sustained release of ART could be further extended from ≈7 to 14 h by increasing the mass of drug‐loaded NFN in the drip chamber (Figure 4c). The release profile could also be changed by an additional PPX coating on the NFN without change of the amount of ART in the NFN (Figure 4d).

Under similar conditions (infusion medium: 0.5 wt% Tween80; flow rate: 0.083 mL min^−1^) the drug release experiment using ART powder in the drip chamber of an infusion system led to not controllable release of ≈30 wt% drug in 15 h. The release curve showed a fast release regime in which about 20 wt% of the drug was released in about 7 h followed by a very slow release. The faster release in the beginning might be due to the dissolution of small ART crystals. The dissolution of hydrophobic drugs is dependent on the crystal size,^25^ smaller crystals dissolve faster than bigger. The release of complete drug was hindered and after about 3 h the degradation products in HPLC were already obvious (compare also Figure S8–S10).

We found a new paradigm for the programmed ART release from nanocarriers, NFN adapted to the infusion system. The ART‐loaded NFNs provide optimal platform for precise programmed drug release when applied in an infusion system, which could also be applied in future for other hydrophobic drugs. For this programmable release profile, NFNs are essential and versatile enough as they provide excellent control of preparation, handling, and mass transfer. Overall, we have decoupled the programmed release of drug from the influence of physiological environment and provided a precise and reproducible drug release with different release profiles for a drug with problems of low bioavailability, low solubility, and instability in an aqueous medium. The controlling handles responsible for a particular release profile are precisely under control through infusion system and eluting medium ART‐loaded NFN, and controlled environmental factors. Another distinct advantage of the infusion system is the lack of contact of the NFN with the body of the prospective patient. Consequently, biocompatibility of the ART carrier becomes an irrelevant issue for its therapeutic application. Also, the concept can be easily extended to the combination of different drugs for therapeutic requirements in a simple way. For example, for malaria treatment, where drug resistance is relevant the combination of artemisinin derivative artemisinin derivative with mefloquine, another hydrophobic drug, might be required for simultaneous application. It is conceivable in the infusion system with the same advantages mentioned above for ART. In conclusion, the integration of drug‐loaded NFN in infusion systems represents a highly versatile and universal paradigm for manifold applications of nanoscale carriers. A realistic perspective for clinical translation of this concept for treatment of malaria is presented.

## Experimental Section

All used materials and experimental methods have been described in details in the Supporting Information.

## Supporting information

As a service to our authors and readers, this journal provides supporting information supplied by the authors. Such materials are peer reviewed and may be re‐organized for online delivery, but are not copy‐edited or typeset. Technical support issues arising from supporting information (other than missing files) should be addressed to the authors.

Supplementary
