# Supplementary material for: Unlocking Nanocarriers for the Programmed Release of Antimalarial Drugs
Source: Glob Chall. 2017 Jan 30;1(2):1600011. doi: 10.1002/gch2.201600011 (PMC6607132; doi:10.1002/gch2.201600011)
Supplement: Supplementary file 1 — Supplementary [file GCH2-1-1600011-s001.pdf]

Copyright WILEY-VCH Verlag GmbH & Co. KGaA, 69469 Weinheim, Germany, 2017.

Global Challenges

Open Access

Supporting Information

for *Global Challenges*, DOI: 10.1002/gch2. 201600011

Unlocking Nanocarriers for the Programmed Release of Antimalarial Drugs

*Amir Reza Bagheri, Seema Agarwal, Jacob Golenser, and Andreas Greiner**

Copyright WILEY-VCH Verlag GmbH & Co. KGaA, 69469 Weinheim, Germany, 2016.

WILEY-VCH

Supporting Information

Unlocking nanocarriers for the programmed release of antimalarial drugs

*Amir Reza Bagheri, Seema Agarwal, Jacob Golenser, and Andreas Greiner**

Supporting information

Unlocking nanocarriers for the programmed release of antimalarial drugs

*Amir Reza Bagheri, Seema Agarwal, Jacob Golenser, and Andreas Greiner**

A. R. Bagheri, Prof. Dr. S. Agarwal, Prof. A. Greiner
Macromolecular Chemistry, Bavarian Polymer Institute, University of Bayreuth,
Universitätsstraße 30, 95440 Bayreuth, Germany
E-mail: greiner@uni-bayreuth.de
Prof. Dr. J. Golenser
Department of Microbiology and Molecular Genetics, The Kuvin Centre for the Study of Infectious and Tropical Diseases, The Hebrew University of Jerusalem, Jerusalem, Israel

Keywords: programmed drug delivery, nanocarriers, electrospun nanofibers, antimalarials,

Materials

ε- Caprolactone (CL) was purchased from Alfa Aesar and was dried over calcium hydride (Merck Co.) for 24 hours and purified by vacuum distillation. α- Hydroxy-ω-methoxy-poly (ethylene glycol) (MPEG, M_w: 5000) from Aldrich Co. was dried out in a vacuum oven at 40 °C for 2 hours. The poly(ethylene oxide) (PEO, M_w: 900,000) was purchased from Acros Co., and it was used in dried form after drying in vacuum oven at 40 °C for 2 hours. Stannous octoate [$Sn(Oct)_2$] and sodium dodecyl sulfate (SLS), Tween 80, pyridine, THF, methylene chloride from Sigma-Aldrich and formic acid from Fluka were used as received. Parylene N was used as received (Specialty Coating Systems, SCS). ART was donated by CIPLA and used without further purification.

Polymer synthesis. The biodegradable diblock copolyester PCL_{16500}-b-$MPEG_{5000}$ (the subscript describes the M_n of the polycaprolactone (PCL) block and of MPEG according to NMR analysis) (PCL-MPEG) was synthesized according to scheme S1, following standard protocols (Bubel et al. 2013; Meier et al. 2005) with some important modifications, by ring-opening of ε-caprolactone catalyzed by stannous octoate in the presence of α-methoxy-ω-hydroxy-poly (ethylene glycol) (MPEG) as a macroinitiator.

The reaction conditions are given in the Table S1. For purification of the polymer, after precipitation in n-pentane, the polymer was extracted with water to remove the water-soluble parts of the product, like the oligo PCL-b-MPEG and unreacted MPEG. The purified polymer showed monomodal GPC curve (Fig. S1). The isolated yields were in the order of 90–92% for the different synthesis attempts. The expected chemical structure of PCL-MPEG was confirmed by observation of characteristic peaks of both MPEG and PCL in the ^{1}HNMR spectrum (Fig. S2, C). $-OCH_2$-(protons of MPEG, 6/6') and PCL (1) were observed at 3.61(s) and 4.03 (t) ppm, respectively. The other protons of PCL were obtained at 2.27 (t) ppm [CH_2CH_2C (O) O-, 5], 1.56−1.67 (t) ppm [$-OCH_2CH_2CH_2CH_2$-, 2; -C (O) $CH_2CH_2CH_2$-, 4], and 1.29−1.40 (m) ppm [C (O)$CH_2CH_2CH_2CH_2CH_2$O-,3] (Fig. S2).

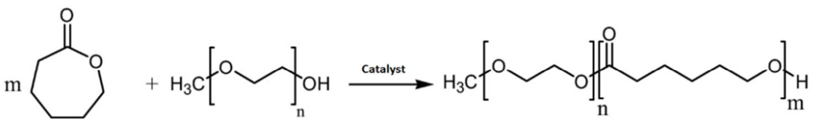

Scheme S1. Formation of block copolymers of polycaprolactone (PCL) and methoxy terminated polyethylene glycol (MPEG).

CL (g)	MPEG (g)	Catalyst (μL)	Time (h)	Temperature (°C)
13.95	4.65	30	3	130

Table S1. Parameters for the synthesis of PCL-MPEG

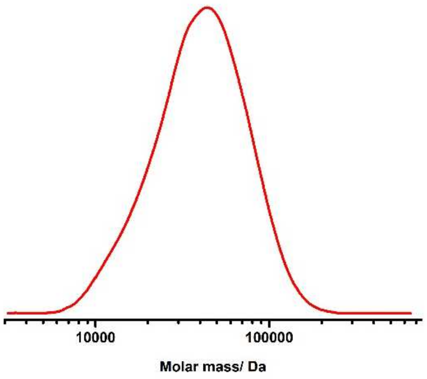

Figure S1. GPC chromatogram of synthesized PCL_{16500}-b-$MPEG_{5000}$

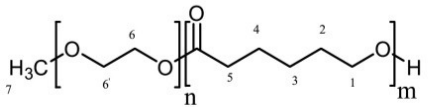

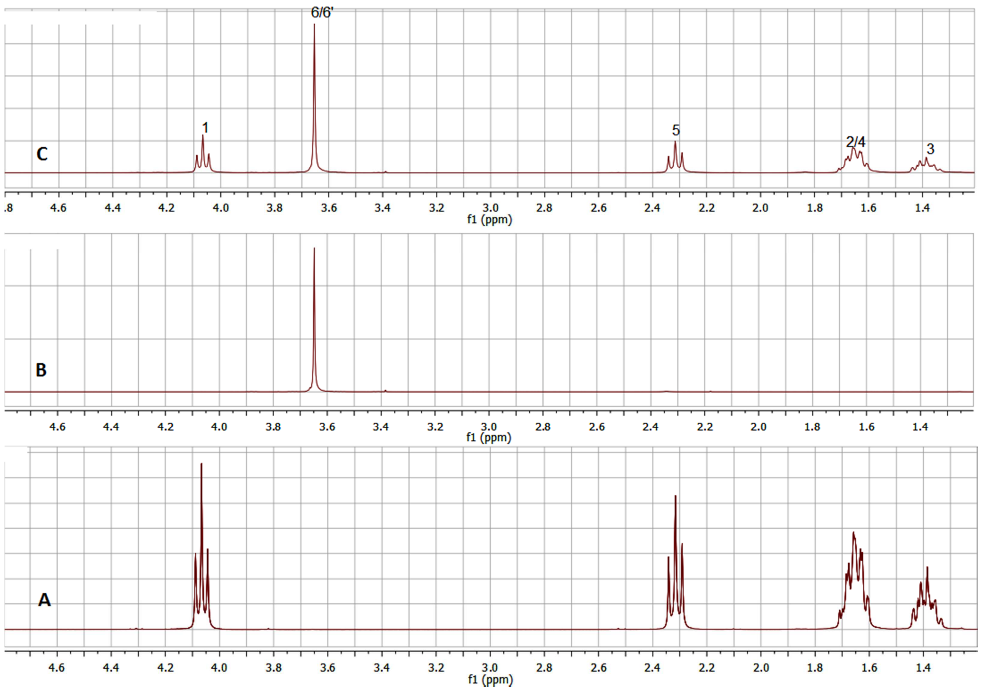

Figure S2. ^{1}HNMR spectra of A. PCL, B. MPEG and C. PCL_{16500}-b-$MPEG_{5000}$

Polymer	M_n (expected)	M_n (^{1}HNMR)	M_n (GPC)	PDI	T_g (°C)	T_m (°C)
**PCL_{16500}-b-$MPEG_{5000}$**	20,000	21,500	32,700	1.44	-56 to -58	60 to 62

Table S2. Data of synthesized polymer

Preparation of ART-loaded NFNs

Drug loaded polymeric solutions composed of 15 wt. % PCL-MPEG, 2.5 wt. % pyridinium format (PF) and different amounts of ART in methylene chloride (MC) were prepared. ART quantities could be variable due to the fabrication of different drug loaded samples with different contents of the drug. PF has been produced by mixing of 1:1 (mol: mol) pyridine and formic acid. The mixtures were protected from light and stirred at least for 5 hours at room temperature to form homogenous solutions. The solutions were electrospun with a voltage of 30 kV at a distance of 15 cm and a flow rate of 1.33 ml. hr^{-1} on aluminium foil collecting electrode. An air humidifier controlled the humidity at 18–25% and the temperature was adjusted to 20-22 °C. PF was added to the electrospinning solution for control of the electric conductivity of the formulation. During electrospinning PF evaporates as pyridine and formic acid and cannot be traced anymore in the deposited ART containing NFN. The increase in the electrical conductivity by the addition of PF caused a significant reduction of bead formation. The integrity of ART after electrospinning was verified by solution NMR.

Identification and quantification of ART in drug loaded NFNs by NMR. NMR spectroscopy was applied to identify and quantify the ART content in drug loaded samples. Different hydrogen atoms in chemical structure of ART could be recognized by this technique (Fig. S3), but two main peaks, first one at δ : 5.3 ppm (1H, s, H-1) and second one at 4.2 ppm (1H, d, H-2) were employed as the most characteristic peaks to follow the presence of ART. A single signal (signal 1. in Fig. S3) at δ: 5.3 is used in this work as the unique peak for quantification of ART content (Tab. S3). For determination of ART content, each sample has been quantified by examining of at least three NFN.

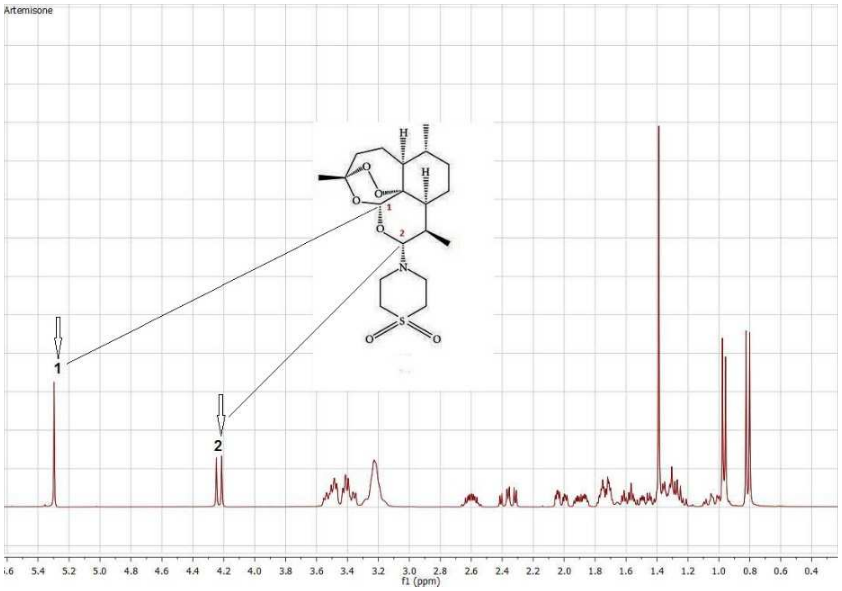

Figure S3. ^{1}HNMR spectrum of pure ART in $CDCl_3$.

ART loaded sample	Theoretical drug content (wt. %)	Integral $_{ART}$ / Integral $_{PCL\text{-}b\text{-}MPEG}$	Experimental drug content (wt. %)	Assay (%) ± SD
NFN-sample 1	14.30	4.15	14.26	99.70 ± 0.5
NFN-sample 2	12.5	3.61	12.41	99.28 ± 0.5

Table S3. Determination of ART content by NMR spectroscopy (solvent: $CDCl_3$)

The calibration line shown in Fig. S4 was obtained by quantitative NMR of mixtures of ART and polymer by comparison of the integrals at δ: 5.3 ppm for ART to δ: 4.03 for PCL-MPEG.

$$\text{Assay (\%)} = \frac{\text{Experimental content of ART in each NFN mat (\%)}}{\text{Theoretical content of ART in each NFN mat (\%)}} \times 100$$

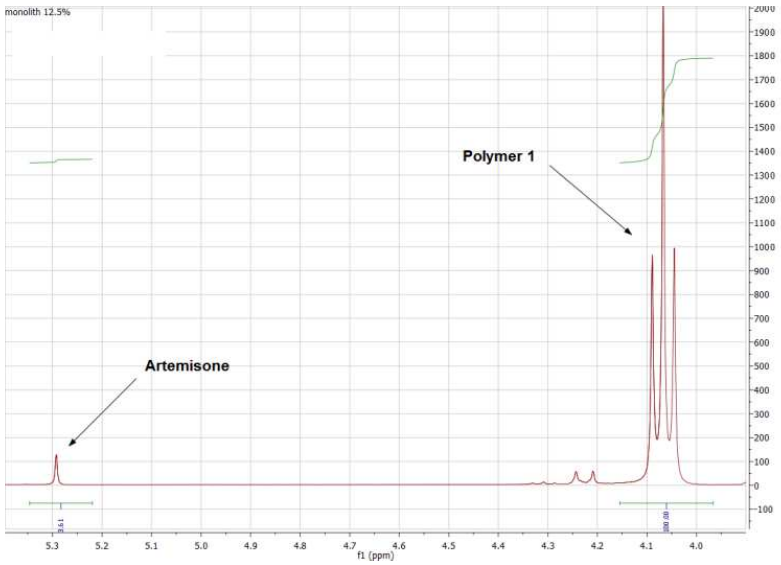

Figure S4. ^{1}HNMR spectrum of ART loaded NFN with the drug content of 12.5 wt. %

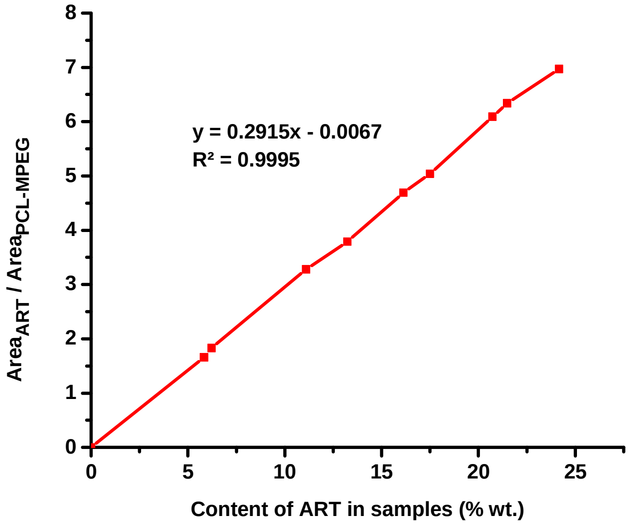

Figure S5. Calibration line of standard solutions of ART and polymer in $CDCl_3$, relative standard deviation (RSD) values were for all experiments below 2%.

Quantification of ART in drug loaded NFNs by HPLC

HPLC instrument was equipped with an Eclipse XDB-C18, 4.6 x 150 mm, 5 µm column. The acetonitrile (50 % v), H_2O (30 % v), and methanol (20 % v) was used as mobile phase with UV detector at λ: 260 nm. The temperature of the column was adjusted to 35°C, the flow rate of the mobile phase was 0.8 ml/min and the injection volume was 20 µl.

The different NFNs were dissolved in mobile phase and encapsulated ART was readily dissolved in solvent after stirring at room temperature for estimation by HPLC. For this purpose, at least, three samples of ART loaded products were dissolved in mobile phase and then analyzed at λ: 260 nm by HPLC (Fig. S6). The assay values were achieved by comparison between data obtained from ART loaded samples and the standard data which were gained from standard solutions. These results were compared with results from quantitative NMR (Table S. 3 and S. 4). This comparison confirmed that all assay data are reproducible and repeatable in both methods.

ART loaded sample	Theoretical drug content (wt. %)	Experimental drug content (wt. %)	Assay (%) ± SD
NFN-Sample 1	14.30	14.24	99.55 ± 0.4
NFN-sample 2	12.5	12.44	99.50 ± 0.7

Table S4. Determination of ART content by HPLC

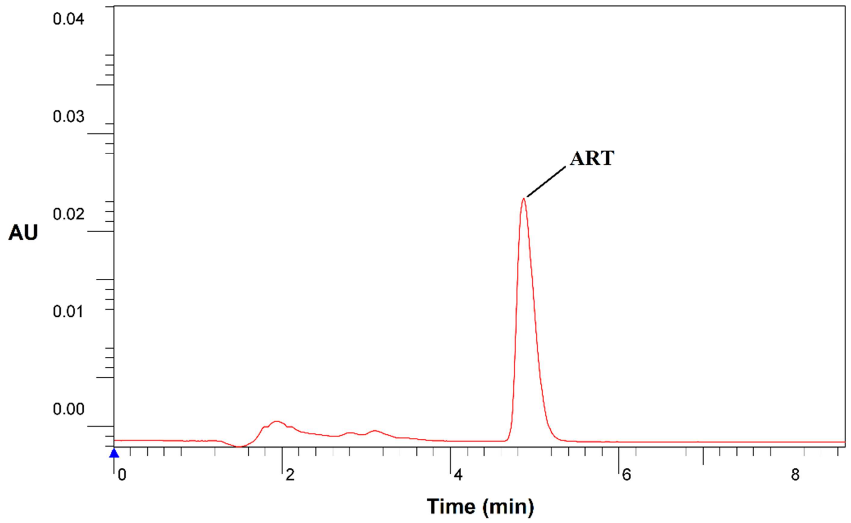

Figure S6. HPLC chromatogram of eluted ART from NFN in the mobile phase

Validation of HPLC method for quantification of ART

The HPLC method was validated by preparation of ART solutions in aqueous medium which contained 1 % w/v SLS in water. The lowest concentration of ART which could be detected by this method was 0.0156 mg/ml (Fig. S7).

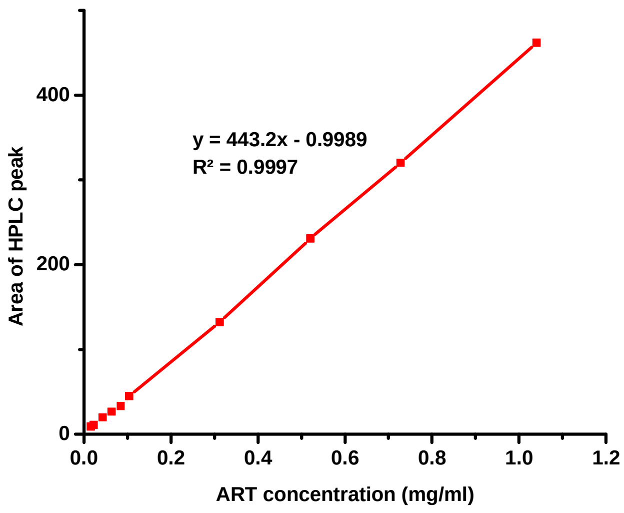

Figure S7. Validation of HPLC method based on repeatability for different solutions. RSD values were for all experiments below 2%.

Preparation of PPX coated NFN

PPX coating was done in lab coater 2010 (SCS) using [2.2] paracyclophane (Parylene N) as a precursor according to Gorham's method. The thickness of the PPX coating was controlled by adjustment of the time of PPX deposition.

Determination of *in vitro* cumulative release of ART from NFN

The weighed drug loaded mats were incubated at 37°C in 10 ml of medium (1 % w/v SLS in water) with pH values of 7.4 ± 0.1. All samples were protected from light. At certain incubation time points, 1 ml of each solution was taken out from vials and replaced with 1 ml fresh medium. The released ART in the medium was determined by a HPLC instrument with the same method which was described. All release results have been reported as mean ± SD.

Degradation of ART in solution

The degradation studies were carried out by putting definite amount of ART powder in 1% w/v SLS at 37 ± 0.5 °C. The amounts of ART were quantified before incubation and then all solutions were stored in a thermostatic incubator at a temperature of 37 ± 0.5 ^{o}C and shaken at 50 rpm. 1 ml of each solution was taken out and, finally, the dissolved amounts of ART were quantified by HPLC method which was described previously. All experiments have been repeated three times and mean data have been reported in this work. Relative standard deviation (RSD) for all experiments were below 2% and they were obviously repeatable. Our long-term studies demonstrated only 14 % ART were remained after 19 days (Fig. S8)

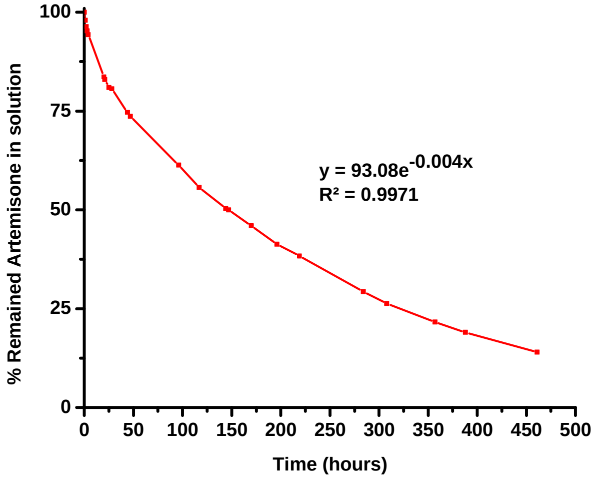

Figure S8. Decomposition of ART in aqueous medium composed of 1 % w/v SLS, pH of medium: 7.4 ± 0.1 and temperature: 37 ± 0.5 ^{o}C. Concentration of ART: 1.03 ± 0.02 mg/ml. All tests were repeated three times. RSD values for all experiments were below 2 %.

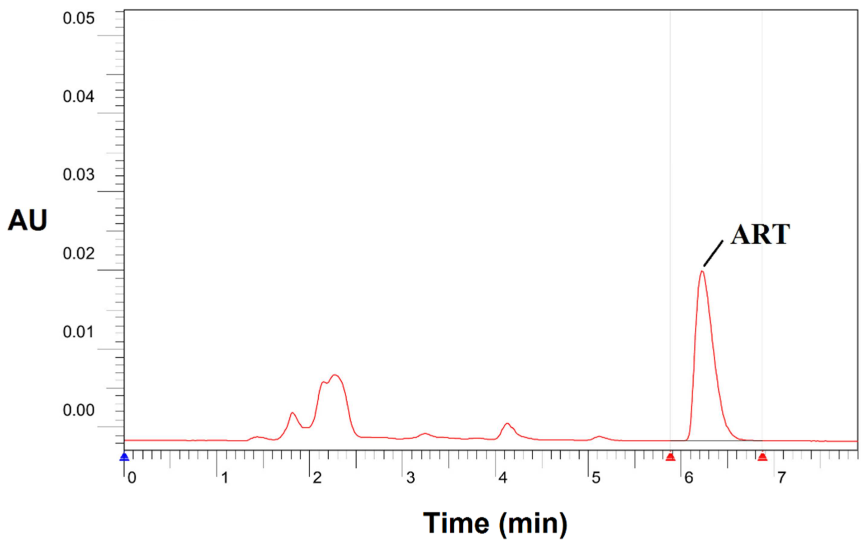

Figure S9. HPLC chromatogram of dissolved ART in aqueous medium containing 1% w/v SLS, concentration of ART in solution: 1.045 mg/ml, fresh solution was prepared at room temperature.

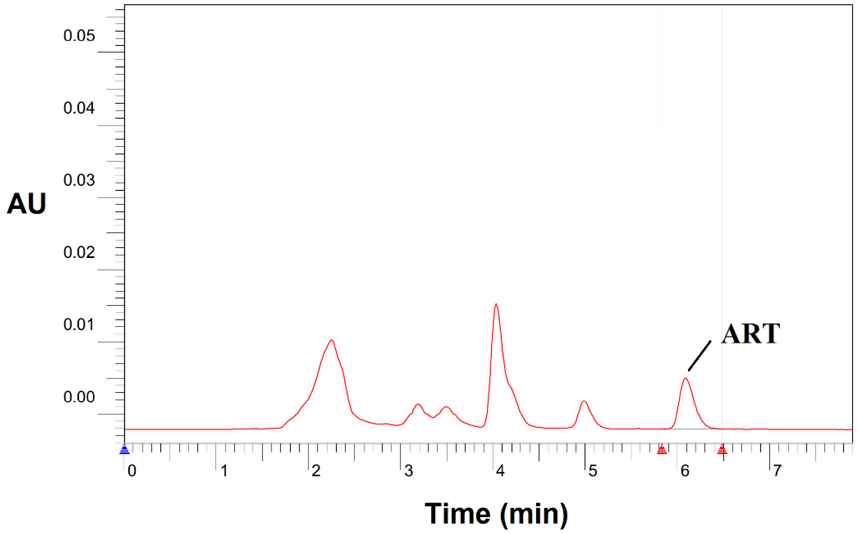

Figure S10. HPLC chromatogram of a solution of ART in aqueous medium containing 1% w/v SLS, concentration of ART in solution: 1.045 mg/ml, the solution was stored in incubator at 37 ± 0.5 ^{o}C for 308 hours (about 13 days).

Set-up for infusion experiments: An infusion pump from B. Braun Melsungen was used together with a standard infusion system as shown in Fig. S11. The drip chamber was opened, equipped with the ART-loaded NFN, and closed again prior to the infusion experiment.

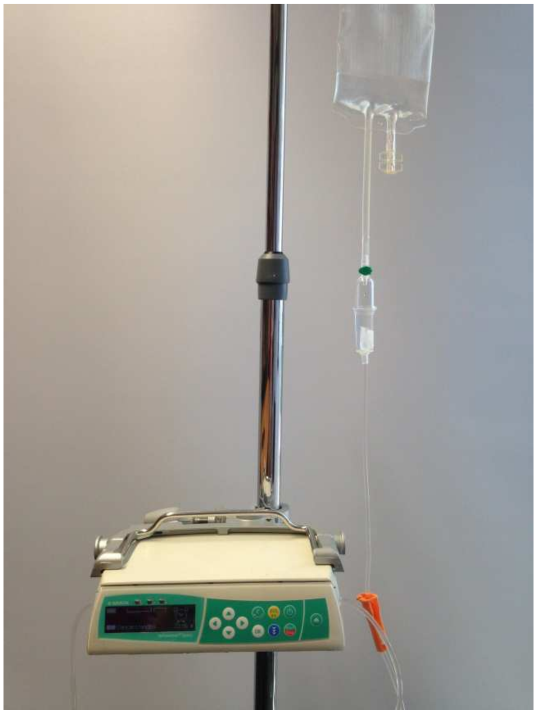

Figure S11. Photo of the infusion set-up used for the infusion experiments with ART-loaded NFN.

Determination of released amount of ART from NFN under infusion conditions

The weighed drug loaded mats were placed in drip chambers of the infusion set and then the drip chambers were connected to infusion solution bottles and filled by physiological media which were 0.9 % w/v saline solution with 0.5 or 1 wt. % Tween 80. NFN in the drip chambers (volume 8 mL) were always covered by infusion medium. The pH values of media were adjusted to 7.4 ± 0.1 by the addition of small amounts of sodium bicarbonate. The flow rates were adjusted before starting the release experiments. The amounts of ART in samples were analysed by HPLC method as previously described. Each experiment was repeated at least three times and the mean ± standard deviations (SD) were reported.

Increase solubility of ART by addition of solubilizers. Our studies confirmed that ART was practically insoluble in water and aqueous buffer systems such as PBS buffer. In addition, ART was degraded during incubation. Therefore, it was vital to overcome these obstacles. We focused on validation of a protocol aiming at improvement of ART solubility in aqueous medium by adding a solubilizer. Consequently, we applied sodium lauryl sulfate (SLS) as a solubilizer for determination of *in vitro* cumulative release and Tween 80 as a biocompatible solubilizer that is used for formulation of the infusion mediums (and increased ART solubility in our experiments, Fig. S12).

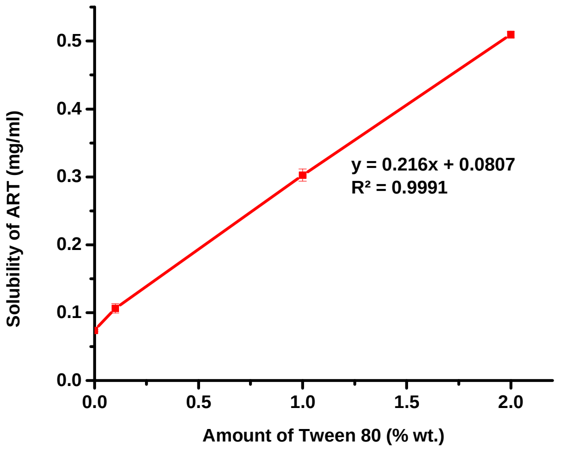

Figure S12. Increasing the solubility of ART in the aqueous media by addition of tween 80 as the biocompatible solubilizer**.**

Instrumental characterization

NMR: Nuclear magnetic resonance (NMR) spectroscopy was conducted in $CDCl_3$ as a solvent using a Bruker ARX300 spectrometer and MestReNova software for evaluation.

GPC: Gel permeation chromatography (GPC) was carried out in THF using a PSS-SDV pre-column (8 mm × 50 mm) and a PSS-SDV column (linear XL, 5 µm, 8 mm × 300 mm) at 40°C

with a flow rate of 1 mL/min and Win GPC Unity (build 6807) for evaluation. The standard calibration was applied by using polystyrene as the standards.

HPLC: High-performance liquid chromatography was performed using Waters system equipped with autosampler AS100 and waters 2489 as UV detector.

PPX coating: All PPX coated samples were fabricated by Labcoater 2010 (SCS) using Parylene N as a precursor.

SEM and EDX Microscopy: Scanning electron microscope (SEM) was conducted using electron microscope model LEO 1530. ImageJ software was applied for evaluation of micrographs.

References

Bubel, K., Zhang, Y., Assem, Y., Agarwal, S., Greiner, A., 2013. Macromolecules Tenside Free Biodegradable Polymer Nanofiber Nonwovens by "Green Electrospinning". 46, 7034-7042.

Meier, M. A. R., Aerts, S. N. H., Staal, B. B. P., Rasa, M., Schubert, U. S., 2005. PEO-b-PCL Block Copolymers: Synthesis, Detailed Characterization, and Selected Micellar Drug Encapsulation Behavior. Macromol. Rapid Commun. 26, 1918-1924.
